# Compost and Crude Humic Substances Produced from Selected Wastes and Their Effects on *Zea mays* L. Nutrient Uptake and Growth

**DOI:** 10.1155/2013/276235

**Published:** 2013-11-06

**Authors:** Perumal Palanivell, Kasim Susilawati, Osumanu Haruna Ahmed, Nik Muhamad Majid

**Affiliations:** ^1^Department of Crop Science, Faculty of Agriculture and Food Sciences, Universiti Putra Malaysia Bintulu Sarawak Campus, 97008 Bintulu, Sarawak, Malaysia; ^2^Institute of Tropical Forestry and Forest Products (INTROP), Universiti Putra Malaysia, 43400 Serdang, Selangor, Malaysia

## Abstract

Production of agriculture and timber commodities leads generation of enormous quantity of wastes. Improper disposal of these agroindustrial wastes pollutes the environment. This problem could be reduced by adding value to them. Therefore, a study was carried out to analyse and compare the nutrients content of RS, RH, SD, and EFB of composts and crude humic substances; furthermore, their effect on growth, dry matter production, and nutrient uptake for *Zea mays* L., and selected soil chemical properties were evaluated. Standard procedures were used to analyze humic acids (HA), crude fulvic acids (CFA), crude humin (CH), soil, dry matter production and nutrient uptake. Sawdust and RS compost matured at 42 and 47 days, respectively, while RH and EFB composts were less matured at 49th day of composting. Rice straw compost had higher ash, N, P, CEC, HA, K, and Fe contents with lower organic matter, total organic carbon, and C/N and C/P ratios. The HA of sawdust compost showed higher carbon, carboxylic, K, and Ca contents compared to those of RS, RH, and EFB. Crude FA of RS compost showed highest pH, total K, Ca, Mg, and Na contents. Crude humin from RS compost had higher contents of ash, N, P, and CEC. Rice straw was superior in compost, CFA, and CH, while sawdust compost was superior in HA. Application of sawdust compost significantly increased maize plants' diameter, height, dry matter production, N, P, and cations uptake. It also reduced N, P, and K based chemical fertilizer use by 90%. Application of CH and the composts evaluated in this study could be used as an alternative for chemical fertilizers in maize cultivation.

## 1. Introduction

The agriculture sector plays very important role in Malaysia and elsewhere. In Malaysia, it contributed US$ 230.83 billion to the gross domestic product in 2008 [4]. The economic contribution is through production of a vast number of agricultural and timber commodities such as oil palm, rubber, paddy, sawn timber, and poultry. According to the Malaysian Palm Oil Board, about 90.048 million metric tonnes of fresh fruit bunches of oil palm was produced in 2009 [5]. In the timber industry, about 1.9 million meter cubes (m^3^) of sawn timber was exported in 2009 [6]. In 2009, about 2,511,043 metric tonnes paddy was produced in Malaysia [7]. According to the Federation of Livestock Farmers' Associations of Malaysia, about 516.23 million birds (broiler) were produced and 43.08 million live birds were exported in 2009 [8, 9]. To sustain production of agricultural commodities, Malaysia imports significant amount of chemical fertilizers annually. Malaysia's total import value of N, P, and K fertilizers in 2008 was US$ 2.96 billion [4].

Production of agriculture and timber commodities leads generation of enormous quantity of wastes such as oil palm empty fruit bunch (EFB), rice straw (RS), rice husk (RH), sawdust (SD), and chicken dung. Most of these wastes are not properly disposed. For instance, RS is usually burned [10] *in situ* after grain harvest. Rice husk and SD are also openly burned or dumped around milling stations. However, EFB is mostly applied in oil palm plantations as mulch [11]. In some cases, the EFB is dumped in plantations to degrade but it takes longer time to do so. By the time EFB degradation completes, it serves as habitat for insects and pests and this causes problems to oil palm plantations [12]. 

Inappropriate disposal of these wastes can cause air, water, and land pollution [13]. As an example, burning of agricultural or organic wastes releases particles [10] and greenhouse gases into the atmosphere which cause several environmental and health problems [14]. Environmental problems associated with inappropriate management of these organic wastes could be reduced through composting [15–17]. Composting can be defined as rapid reduction of large volumes of organic materials through biological process [18]. Utilization of organic wastes also reduces excessive use of chemical fertilizers. Furthermore, it reduces eutrophication due to leaching and deposition of nutrients from chemical fertilizers to water bodies [19, 20]. Composts generally improve soil fertility by playing essential role in improving soil physicochemical and biological properties. Besides conditioning soils, they serve as slow release fertilizers during mineralization compared to mineral fertilizers such as urea, muriate of potash, and triple superphosphate, known for being highly soluble upon soil application. Hence, they are used as an alternative to conventional fertilizer to increase crop production.

Composting of these agroindustrial wastes may produce composts which are rich in humic substances and nutrients through humification and mineralization [3, 21–23]. Humic substances are heterogeneous organic macromolecules, consisting of humic acids (HAs), fulvic acids (FAs), and humin. Crude humin in this study refers to unpurified humin. Humic susbtances improve soil fertility through improvement of soil physiochemical properties *via* improvement of soil structure, as source of nutrients and trace minerals for plant uptake with induced microflora and fauna activities which are important in the life cycle on the earth. Furthermore, they affect physiological, metabolical, and developmental processes of plants. Additionally, humic substances cause activation of plasma membrane H^+^-ATPase, respiration, and activation of genes involved in nitrate (NO_3_^−^) intake in plants. Studies have shown that high and low molecular weight fractions of humic substances promote stomatal opening. Besides increasing soil organic matter composition, they play major factor in environmental recovery through phytoremediation and vegetation revival in infertile soil [24, 25]. 

Although use of composts as organic fertilizer [26] is well known, only few studies have been conducted on crude humins as plant nutrients. Besides HA and FA, a study has shown that addition or application of crude humins from composted sago waste can increase plant dry matter production, nutrient uptake, and use efficiency [27]. 

Thus, in this study, the nutrient contents of RS, RH, SD, and EFB of composts and crude humic substances were analysed and compared; furthermore their effect on growth, dry matter production, nutrient uptake for *Zea mays* L., and selected soil chemical properties were evaluated.

## 2. Materials and Methods

The RS was sampled in a paddy field of Universiti Putra Malaysia Bintulu Sarawak Campus, Malaysia. Rice husk was collected from Rumah Serit, Katibas, Ulu Kapit, Sarawak, Malaysia. Oil palm empty fruit bunch was obtained from Lambir Estate, Sarawak Oil Palm Berhad, Miri, Sarawak, Malaysia. Sawdust was collected from Ling Brothers Sdn. Bhd., Kemena Commercial Center, Jalan Sungai Nigu, Bintulu, Sarawak, Malaysia. These wastes were air-dried and ground using Retsch SM100 Comfort Cutting Mill to reduce the size. Composting of RS, RH, SD, and EFB was carried out in a 48 × 35.5 × 34.7 cm sized white polystyrene box. The study had the following treatments:

RS: rice straw (75%) + chicken dung (15%) + molasses (6%) + urea (2%) + rock phosphate (2%),
RH: rice husk (75%) + chicken dung (15%) + molasses (6%) + urea (2%) + rock phosphate (2%),
SD: sawdust (75%) + chicken dung (15%) + molasses (6%) + urea (2%) + rock phosphate (2%),
EFB: empty fruit bunch (75%) + chicken dung (15%) + molasses (6%) + urea (2%) + rock phosphate (2%).

Each treatment was replicated three times in a completely randomized design. Prior to composting, each mixture was moistened using the tap water up to 50 to 60% moisture content and this moisture was maintained throughout the composting period. Ambient and compost temperature were taken daily (8 a.m. and 5 p.m.) using a digital thermometer (Checktemp M-28390, HANNA instruments). The compost temperature was monitored until it was equivalent to ambient temperature and turning was done when necessary. The compost mixture (before composting), composts, and crude humins were analyzed for pH [28], total nitrogen [29], organic carbon and organic matter content [30], CEC [31], and HA [32, 33]. Total cations and P were extracted using the dry ashing method [30]. Cations content was determined using atomic absorption spectrophotometry (AAnalyst 800, Perkin Elmer Instruments, Norwalk, CT) and P content was determined using the Blue Method [34].

The isolation of HA was done using the method of Ahmed et al. [32] and Palanivell et al. [33], with some modification. The compost and 0.1 M KOH solution were placed in polyethylene centrifuge bottle at a ratio of 1 : 10 (w/v). The mixture was shaken at 180 rpm for 24 hours at room temperature (approximately 25°C). The mixture was centrifuged for 15 min at 10,000 rpm. The dark-coloured supernatant liquid (mixture of crude humic acids and fulvic acids) was decanted and filtered using Whatman filter paper number 2. Solid residue (crude humins) remaining in the bottle was collected and air-dried for analysis. The pH of the supernatant liquid (mixture of humic and fulvic acids) was adjusted to 1.0 using 6 M HCl and left at room temperature for at least 3 hours. The suspension was transferred into a polyethylene centrifuge bottle and centrifuged at 10,000 rpm for 10 minutes. The HA was purified 5 times as described by Ahmed et al. [32] and Palanivell et al. [33], using distilled water. Afterwards, it was centrifuged at 10,000 rpm for 10 min to reduce mineral content and HCl during acidification. After the purification, the HA was oven-dried at 40°C until constant weight was attained.

Infrared (IR) spectra of the crude humin and HA were recorded on KBr pellets (1 mg of crude humin or HA plus 100 mg of dry FTIR grade KBr) from 4000 to 400 cm^−1^ on a Thermo Scientific Nicolet 380 FTIR spectrophotometer [35]. Humic acid was characterized for *E*_4_/*E*_6_ (*E* stands for coefficient of extinction) using the method of Campitelli and Ceppi [36] and analyzed using UV-Vis spectrophotometer (PerkinElmer Lambda 25). Total ash and organic carbon contents of HA were determined using the dry combustion method [30]. Humic acid functional group analysis was done according to the method of Inbar et al. [37]. A 20 mg of HA was dissolved in 4 mL of 0.08 M NaOH and shaken for 30 min at 180 rpm. The solution was titrated using 0.01 M HCl to pH 2.5 within 15 min. Phenolic content was measured based on the amount of acid required to titrate the solution from pH 10 to pH 8 and it was assumed that 50% of the phenolic group dissociated from pH 10 to pH 8 [38]. Carboxylic content was calculated based on the amount of acid required to titrate the solution from pH 8 to pH 2.5 and the total acidity was calculated by the summation of carboxylic and phenolic content. 

Crude FA was filtered using Whatman filter paper number 2 prior to analysis. Crude FA was analyzed for pH [28] using a glass electrode and total cations using Atomic Absorption Spectrometer (PerkinElmer AAnalyst 800). Although all the crude humic substances (humic acids, crude fulvic acids, and crude humins) were characterized in this study only crude humins were used in the pot trial. This was because the effects of humic and fulvic acids on plant growth have been extensively studied. 

The soil used in this pot trial was Bekenu series with Ochric Epipedon (Typic Paleudults). The soil was sampled at 0 to 25 cm in an undisturbed area of Universiti Putra Malaysia Bintulu Sarawak Campus, Malaysia, using an auger, air-dried, crushed, and sieved to pass a 5 mm sieve for the pot trial, but for physicochemical analysis, the soil was ground to pass a 2 mm sieve. The soil was analyzed before and after the pot trial. Soil texture was determined using hydrometer method [39]; pH in distilled water and 1 M KCl (at ratio of 1 : 2.5 soil : water or KCl) using a glass electrode [28]; organic matter (OM) and total carbon using loss-on-ignition method [40]; total N using Kjedahl method [29]; available NO_3_^−^ and exchangeable NH_4_^+^ using Keeney and Nelson [41] method. The soil exchangeable cations and available P were extracted using the double acid method [42], after which the cations were determined using Atomic Absorption Spectrometer (PerkinElmer AAnalyst 800). Available P was determined using the Blue Method [34]. Soil CEC was determined using the leaching method [31] followed by steam distillation [29]. The selected chemical and physical properties of the soil used in this study (Table 1) were typical of Bekenu series (Typic Paleudults) and they were consistent with those reported by Paramananthan [1] except for CEC, exchangeable Ca, Mg, and Na. 

The quantity of soil used in the pot trial was determined based on its bulk density and pot size {25 cm (top diameter) × 21 cm (bottom diameter) × 21 cm (height)}. About 8 kg of air-dried soil was weighed into pots. This study was carried out in a temporary rain shelter structure at Universiti Putra Malaysia Bintulu Sarawak Campus which had an average temperature of 31.2 ± 2.1°C, relative humidity of 69.0 ± 14.8%, and light intensity of 964.7 ± 177.9 lux. The pots were arranged in a randomized complete block design (RCBD) with 4 replications. Ten treatments involving crude humins from composts and untreated composts were used in this study (Table 2). Maize seeds (var. Aurora 2 F1 hybrid) were soaked for 24 h in water for better germination and the soil was moistened up to 70% field capacity using tap water for 24 h before sowing. After 24 h, five maize seeds were sowed in each pot at 3 to 4 cm soil depth. Seven days after seeding (DAS), the seedlings were thinned to one seedling per pot to reduce competition between plants.

Fertilizer requirement for the maize crop (60 kg ha^−1^ N, 60 kg ha^−1^ P_2_O_5_, and 40 kg ha^−1^ K_2_O) [2] was scaled down to per plant basis equivalent {Urea (4.84 g plant^−1^), Egyptian Rock Phosphate (ERP) (7.45 g plant^−1^) and Muriate of Potash (MOP) (2.48 g plant^−1^)}. The amounts of CH (T2, T3, T4, and T5) and composts (T6, T7, T8, and T9) were applied based on potassium content. For treatments with crude humins and composts, the amounts of urea and ERP used were reduced because the estimation was based on nitrogen and phosphorus contents in the crude humins and compost. Prior to fertilizer application, the fertilizers were weighed separately and mixed in a 250 mL conical flask using an orbital shaker at 200 rpm [43] for 30 min [44]. For T1 (normal fertilization), the fertilizers were split into two equal applications, that is, at 10 DAS and 28 DAS (conventional practice). For T2 to T9, the fertilizers were applied at 10 DAS only. The plants were monitored up to tasselling stage (48 DAS). This was because this stage is the maximum growth stage of the plants before they enter productive stage [45]. Growth performance in terms of plant height was determined using a measuring tape whilst stem diameter was measured at 10 cm above soil surface using a digital vernier caliper at 48 DAS.**   **Harvesting was done on the 48th DAS. Plant samples were oven-dried at 60°C until constant weight was attained. Prior to analysis, the oven-dried samples were ground using a grinder. Total N was determined using Kjedahl method [29]; selected cations and P were extracted using dry ashing [30]. Cations were determined using Atomic Absorption Spectrometer (AAS) (PerkinElmer AAnalyst 800) while P was determined using the Blue Method [34]. Nitrogen, P, and selected cations concentration in plants were used to calculate nutrient uptake.

Analysis of variance was used to detect significant differences among treatments, while Tukey's test was used to compare treatment means. For the statistical analysis, Statistical Analysis System version 9.2 was used [46].

## 3. Results and Discussion

**Figure 1:**
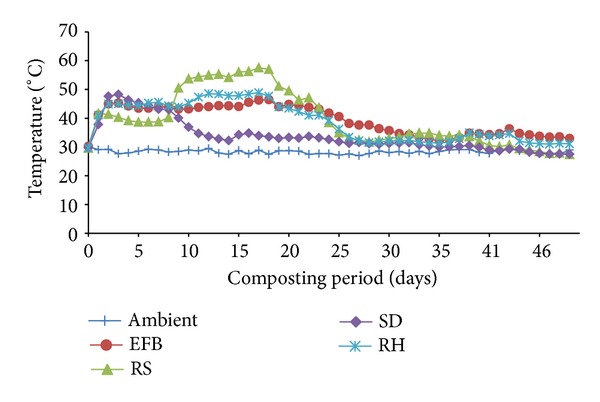
Composts and ambient temperature with time of selected wastes.

All the composts underwent mesophilic, thermophilic, and curing stages (Figure 1). EFB, RH, and RS composts mesophilic phase lasted for 16, 12, and 9 days, respectively. Sawdust compost underwent short mesophilic phase for about 4 days and it had the longest curing phase. Population and diversity of microorganisms vary with different composting stages. Mesophilic microorganisms are active at 40 to 45°C [47]. These microorganisms degraded or used the easily degradable substrate like sugar from molasses, N from urea, and P from ERP for their metabolisms and reproduction. This explains why the compost temperature was higher than that of ambient temperature. 

All the composts reached thermophilic stage (≥45°C) [48]. Rice straw compost showed the highest temperature (57.5°C) and this thermophilic phase lasted for 14 days compared to those of RH, SD, and EFB composts whose thermophilic phase lasted for 11, 4, and 9 days, respectively. Thermophilic stage is very essential during composting as it sanitizes composts by killing pathogens [49]. The longer thermophilic phase shown by RS compost improved the quality of RS compost through rapid degradation of cellulose and lignin [50]. This resulted in higher amount of HA in this compost. Sawdust compost showed the shortest thermophilic stage and this was because of higher lignin content [51, 52]. Sawdust and RS composts took 42 and 47 days, respectively to mature. Rice husk and EFB composts were relatively less matured at the 49th day of composting. Compost maturity is indicated by no more heat production in compost upon several turnings [53].

pH, N, P, CEC, HA, and cations (K, Ca, Mg, Na, Cu, Zn, Fe, and Mn) contents increased at the end of composting. Organic matter, total organic carbon content, C/N, and C/P ratios reduced after composting. Rice straw compost showed higher N, P, CEC, HA, K, and Fe contents with lower organic matter, total organic carbon, and C/N, and C/P ratios compared to other composts. Sawdust compost showed higher organic matter, total organic carbon content, and C/N and C/P ratios but showed lower N, P, K, and Mn contents (Tables 3 and 4). During aerobic composting, C from raw materials is converted to CO_2_ and released to atmosphere. Some of the C may have formed stable carbon compounds such as HA and FA during humification [54, 55]. During humification, organic matter and C reduce while cations, N, and P contents increase [56, 57]. Humification increases cations and CEC because of decomposition of carbon and release of minerals from the carbon matrix. Production of HA during composting increases functional groups such as carboxylic, phenolic, and hydroxylic in composts. These functional groups play important role as exchange site for cations. Higher HA at the end of composting suggests that the compost was mature and stable [36]. The rice straw compost was well decomposed compared to others because it showed higher N, P, cations, CEC, and HA. On the contrary, sawdust mineralized less at the end of composting, hence the associated higher C/N and C/P ratios.

**Figure 2:**
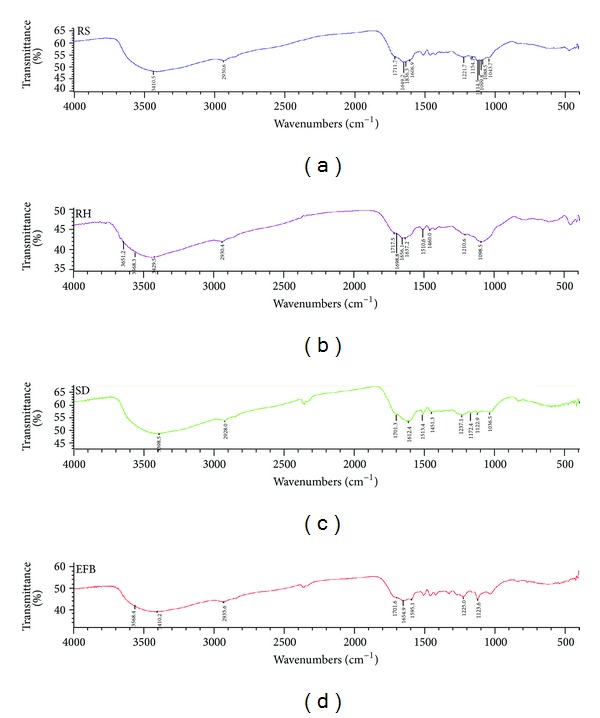
Fourier transform infrared (FTIR) spectra of HA from rice straw, rice husk, sawdust, and oil palm empty fruit bunch.

The infrared spectra (indicating spectral characteristics of HA) of HA are shown in Figure 2. Generally, all the HA showed bands at 3400 cm^−1^ (OH and N–H stretch), 2920 cm^−1^ (aliphatic CH stretch), 1720–1700 cm^−1^ (C=O stretch of carboxylic acid), 1650 cm^−1^ (C=O stretch of primary amide, aromatic C=C, hydrogen bonded C=O, double bond conjugated with carbonyl, and COO– vibrations), and 1240–1154 cm^−1^ (aromatic C–N in plane bend, tertiary amine with C–N stretch, and P–O–C stretch of aromatic phosphates). Humic acids extracted from RS and EFB composts showed 1595 cm^−1^ band (aromatic ring or aryl stretch, N–H bend of secondary amine, carboxylate), whereas 1510 cm^−1^ band (aromatic ring stretch of para- and ortho-disubstituted) was present only in the HA composts of SD and RH. Humic acids isolated from RH and SD composts showed band at 1460 cm^−1^ (aliphatic –CH, –CH_2_, –CH_3_ stretch). Band at 1120 cm^−1^ (C–O stretch of polysaccharides) was present only in HA of RS, SD, and EFB. Humic acids from RS, RH, and SD composts showed bands at 1040–1089 cm^−1^ (C–O stretch of aromatic ether, hydrated polyols, and carbohydrates) [58–61].

The *E*_4_/*E*_6_ (optical density) is the absorbance at two arbitrary selected wavelengths (extinction at 465 and 665 nm). *E*_4_/*E*_6_ value indicates humification level of HA and FA. The HA of RS compost showed the highest *E*_4_/*E*_6_ value. It has been found that the higher the *E*_4_/*E*_6_ ratio of HA, the lower the humification level, molecular weight, and condensation of aromatic compounds [62, 63]. The *E*_4_/*E*_6_, carbon, phenolic, carboxylic contents, and total acidity were within standard range (Table 5). Although all the composts HA chemical properties were within standard range, SD compost HA was better in terms of quality. Humification of SD compost HA was comparably higher compared to HA of other composts. This higher humification level was due to higher lignin content in SD [64]. This observation is supported by the lingo-protein theory or lignin theory [3], which explains synthesis of humic substances. A research by Chefetz et al. [65] has revealed substantial amounts of lignin, protein, and cuticular materials in HA structures using ^13^C-NMR and thermochemolysis-gas chromatography/mass spectrometry techniques. Besides, HA of SD compost showed higher C, carboxylic, total acidity, and cations. Higher total acidity, reflects higher CEC of HA [3, 66, 67].

Among the crude FA, FA of RS compost showed the highest pH, K, Ca, Mg, and Na contents (Table 6). This was due to higher humification and mineralization of RS compost. Higher K content in all crude FAs was because of the KOH used in extracting HA. Other than K, crude FAs also contained Ca, Mg, and Na. Crude humins from RS compost showed higher contents of ash, N, P, and CEC compared to those of CH of RH, SD, and EFB compost (Table 7). This may be due to higher humification and mineralization of RS compost. Crude humins from SD compost were higher in organic matter and total organic carbon. Lower humification and mineralization in SD compost produced crude humins with higher organic matter and total organic carbon. Although all of the crude humins were alkaline, that of EFB compost showed highest pH. Crude humins from EFB compost were higher in K, Cu, Zn, and Ca compared to those of other crude humins. Rice straw and EFB compost crude humins had higher contents of Ca and Mg with significant amount of exchangeable K compared to those of RH and SD compost (Table 8). Hence, highly composted or humified and mineralized compost produces better quality compost and humins in terms of nutrients and CEC.

**Figure 3:**
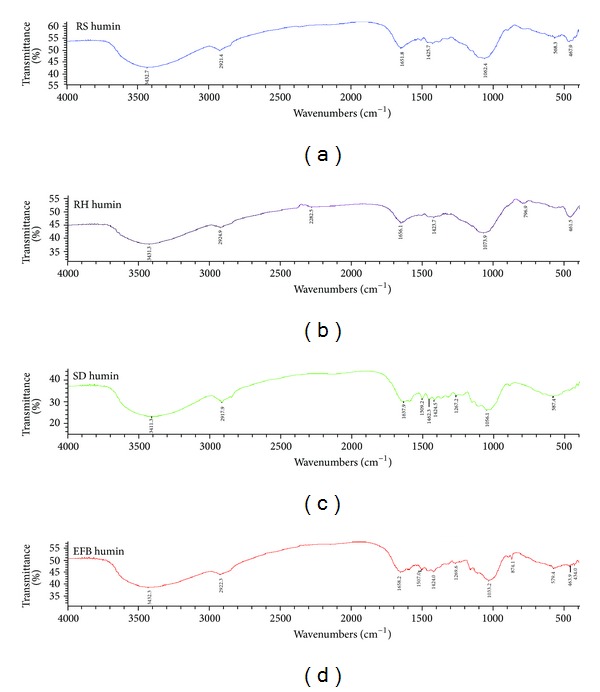
Fourier transform infrared (FTIR) spectra of crude humins from rice straw, rice husk, sawdust, and oil palm empty fruit bunch.

The infrared spectra of crude humins are shown in Figure 3, where different crude humins showed different bands. All crude humins showed bands at 3433–3410 cm^−1^ (OH and N–H stretch), 2925–2917 cm^−1^ (aliphatic CH stretch), 1658–1637 cm^−1^ (C=O stretch of primary amide, aromatic C=C, hydrogen bonded C=O, double bond conjugated with carbonyl and COO^−^ vibrations), 1426–1423 cm^−1^ (C–H bending), and 1033–1074 cm^−1^ (C–O stretch of aromatic ether, hydrated polyols, and carbohydrates). Bands at 2282 cm^−1^ (aliphatic cyanide/nitrile) and 796 cm^−1^ (aliphatic chloro compounds, C–Cl stretch) were only present in crude humin isolated from RH. Crude humins isolated from SD and EFB composts showed bands at 1507–1509 cm^−1^ (aromatic ring stretch of para- and ortho-disubstituted) and 1270–1267 cm^−1^ (C–O stretch, aromatic C–O, C–O ester linkage, and phenolic C–OH). The 1462 cm^−1^ band (aliphatic –CH, –CH_2_, –CH_3_ stretch) was present in only crude humin of SD compost. Crude humins from RS, SD, and EFB composts showed bands at 590–580 cm^−1^ (aliphatic iodo compounds, C–I stretch). Bands 468–463 cm^−1^ (aryl disulfides, S–S stretch) were present in crude humins of RS, RH, and EFB [58–61].

**Figure 4:**
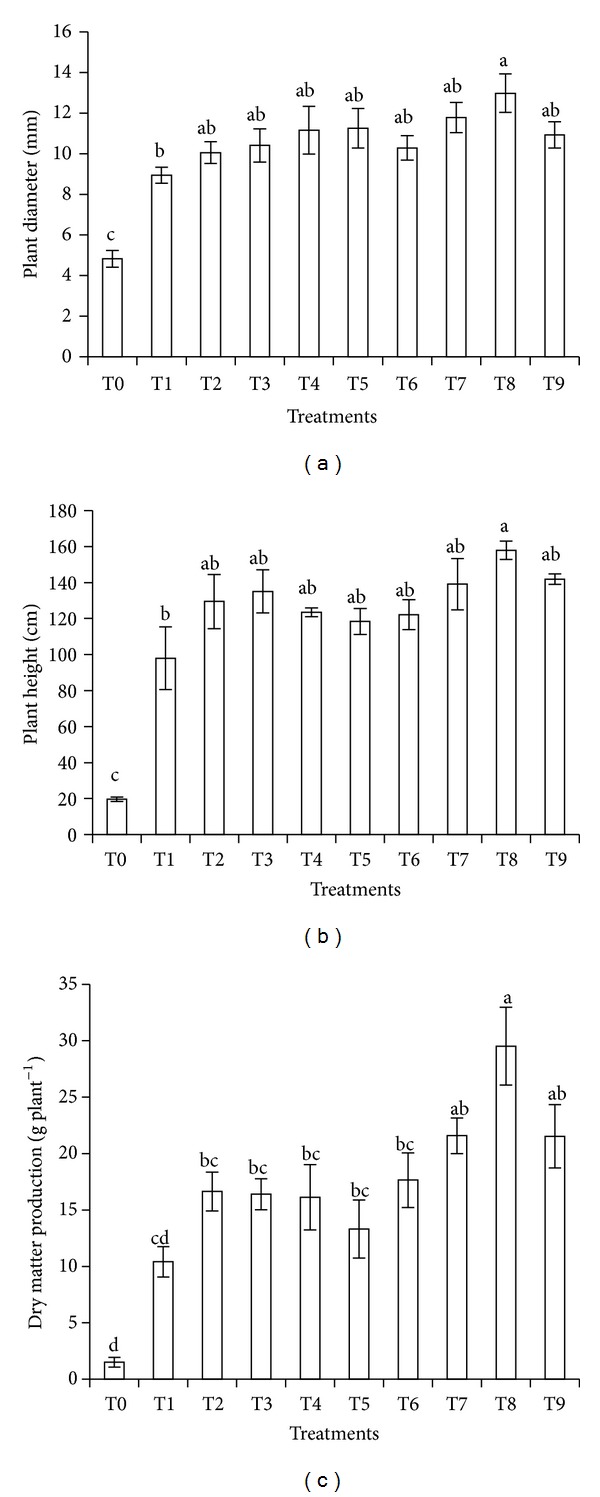
Effect of treatments on diameter, height, and total dry matter production of maize plant at 48 DAS. Different alphabets indicate significant difference between means using Tukey's test at *P* ≤ 0.05.

Treatments effects on maize plant height, diameter, and total dry matter production at 48 DAS are shown in Figure 4. Only plants treated with SD compost (T8) showed greater plant diameter, height, and total dry matter production compared to conventional chemical fertilizer (T1) and without fertilizer (T0). Application of RH (T7) and EFB (T9) composts had significant effect on total dry matter production compared to the conventional chemical fertilizer (T1). T1, T2, T3, T4, T5, T6, T7, and T9 had no significant effect on maize plant diameter and height. In terms of dry matter production, treatments with crude humins (T2, T3, T4, and T5) and compost (T6) showed similar effect as compared to the conventional fertilizer (T1). Maize planted in unfertilized soil (T0) was stunted. This was because of nutrients deficiency in soil to support plant nutrient uptake, growth, and development. 

Significant effect of SD compost (T8) on diameter and height resulted in significant increase of total dry matter production. Composts with low density [68] function as bulking agent and hence they improve soil structure by loosening it and increase the porosity for aeration and root penetration in soils [69]. This may have enhanced maize root penetration and aeration in the rhizosphere. Good roots growth enables them to absorb water and essential nutrients from soil solution to support and increase the crop's growth and development. Composts also provide additional macro- and micronutrients which are very essential for better plant growth.

**Figure 5:**
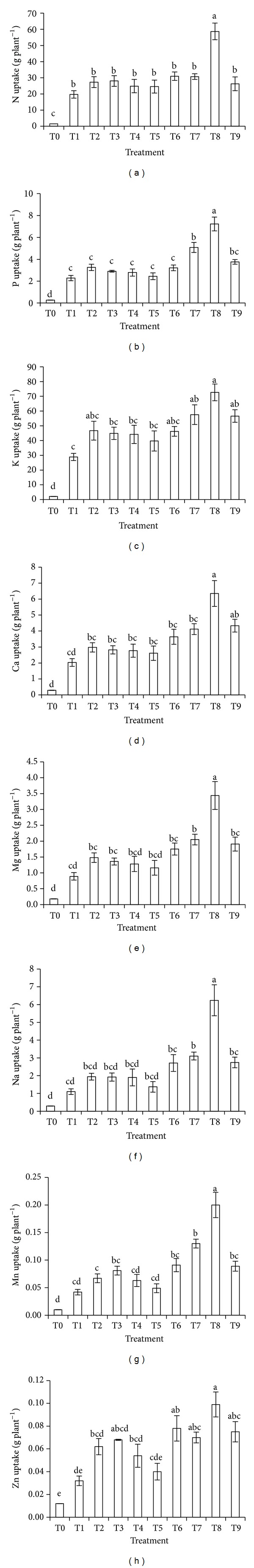
Treatments effects on N, P, K, Ca, Mg, Na, Mn, and Zn uptake of maize plant at 48 DAS. Different alphabets indicate significant difference between means using Tukey's test at *P* ≤ 0.05.

Effects of treatments on N, P, K, Ca, Mg, Na, Mn, and Zn uptake of maize plants at 48 DAS are shown in Figure 5. Plants with SD compost were superior in N and P uptake compared to other treatments. However, application of composts (T6, T7, and T9), crude humins (T2, T3, T4, and T5) and conventional chemical fertilizer (T1) showed similar effect on N uptake. Plants with RH compost (T7) and SD compost (T8) showed higher P uptake compared to the conventional chemical fertilizer (T1). Rice husk compost (T7), SD compost (T8), and EFB compost (T9) significantly increased K uptake compared to conventional chemical fertilizer (T1). 

Sawdust compost (T8) increased Ca, Mg, Na, Mn, and Zn uptake compared to conventional fertilizer (T1) (Figure 5). This was so because application of SD compost (T8) increased organic matter, total carbon (TC), and CEC of the soil (Table 9). EFB compost (T9) had greater effect on Ca and Zn uptake compared to T1. Moreover, RH compost (T7) showed higher total Mg, Na, Mn, and Zn uptake compared to T1 (conventional chemical fertilizer). This may be due to higher organic matter in the compost. Phenolic, carboxylic, alcoholic, and ketonic functional groups are rich in organic matter [70]. These functional groups serve as exchange site and hence increase CEC. Application of chemical fertilizers with composts (which are rich in organic matter) leads to absorption of nutrients at exchange sites. Hence, in this study the composts may have increased retention and release of nutrients slowly in the soil solution for efficient plant uptake. This also plays an important role as a slow release fertilizer [71] by preventing ammonia volatilization and nutrient immobilization. 

Most chemical fertilizers (compound or straight fertilizers) supply only particular nutrients, but composts which are rich in macro- and micronutrients can supply various exchangeable cations. Previous studies had shown that composts can supply nutrients such that, they can be used as an alternative of chemical fertilizers [72]. Besides, addition of composts, vermicomposts, and humates to commercial horticultural potting medium [73] and soil enhanced plant growth, dry matter production, and nutrient use efficiency in tomato [73] and maize plants [27, 74]. Although both NH_4_^+^  and NO_3_^−^  are plant-available forms, NO_3_^−^  is more mobile and plants can absorb it easily [75–77]. The highest available NO_3_^−^  content in sawdust compost (T8) (Table 9) could be one of the reasons why plants grown in T8 showed the highest N uptake compared to T1 (chemical fertilizer). Maize plants treated with SD composts (T8) showed greater N, P, K, Ca, Mg, Na, Mn, and Zn uptake compared to those of conventional fertilizer (T1). A previous study showed that HA application at a rate of 1 g kg^−1^ soil increased nutrient uptake in plants [78] and this observation was consistent with that of T8 where application of SD compost supplied 1.2 g HA kg^−1^ soil. This might be one of the reasons why T8 had greater effect on N, P, and cations uptake. 

Higher contents of carboxylic, phenolic, hydroxylic, and other functional groups in HA and FA function as nutrients chelator [79–81]. Moreover, HA and FA had higher total acidity (CEC) that enables nutrients retention at the exchange site (functional groups) and their timely release for plant uptake. This process reduces NH_3_ volatilization and nutrient leaching. FA has high affinity for mineral chelation and plant growth. They can readily enter plant parts (roots, stems, and leaves) because of their smaller molecular weight and high exchange capacity compared to HA and humins. These allow FA to carry minerals (macro- and micronutrients) into plant parts as they enter into plant tissues. This process increases nutrient uptake and nutrient use efficiency [58, 82, 83].

In general, application of CH (T2, T3, T4, and T5) showed similar effect on maize plant diameter, height, dry matter production, and nutrient uptake compared to conventional fertilizer (T1). This may be due to the absence of HA and FA in CH. Crude humins are also chemically inert [21]. However, it suggests that CH can be used as fertilizer and as an alternative to chemical fertilizer in particular, since CH have similar effect on maize plant as conventional chemical fertilizer.

Selected soil chemical properties at 48 DAS are shown in Table 9. Addition of CH (T2, T3, T4, and T5) and composts (T6, T7, T8, and T9) significantly increased soil pH and exchangeable Mg at 48 DAS. Rice husk and SD composts (T7 and T8) significantly increased soil OM, TOC, and exchangeable Na compared to T1 (conventional chemical fertilizer). Sawdust compost (T8) had significant effect on total N and available NO_3_^−^  compared to T1, but the exchangeable NH_4_^+^ was the highest compared to other treatments. Rice straw CH (T2), RS compost (T6), RH compost (T7), and SD compost (T8) increased soil CEC compared to T1. 

Increase in pH of soils treated with CH (T2, T3, T4, and T5) and composts (T6, T7, T8, and T9) could be attributed to liming effect of these treatments. Thus, usage of lime could be reduced. Higher organic matter content in CH and composts may have played important role in soil buffering capacity as higher organic matter content improves soil buffering capacity and pH [84]. At 48 DAS, CH and composts increased soil exchangeable cations compared to T1. This was because of their richness in these cations. Application of composts (organic matter rich materials) at higher amounts (164.46 g of rice husk compost in T7 and 227.48 g of sawdust compost in T8) increased soil OM and TC contents compared to T1. Soil exchangeable NH_4_^+^ in treatment T1 was the highest because of higher amount of urea used. Nitrogen in the form of NH_4_^+^  ion from urea is more readily available due to its higher solubility. Soil with SD compost (T8) showed higher total N and available NO_3_^−^. This may have released N slowly for a long period of time. Higher organic matter content in CH and composts partly explains why soil treated with RS CH (T2), RS compost (T6), RH compost (T7), and SD compost (T8) showed higher CEC compared to T1. Organic matter, FA, and HA in CH and composts are rich in functional groups and these functional groups serve as exchange sites in soils [85].

## 4. Conclusion

Rice straw produced superior compost because of good humification. It also produced good quality crude fulvic acids and crude humins. However, sawdust compost produced high quality HA. Application of sawdust compost (T8) significantly increased maize plant diameter, height, dry matter production, and N, P, and selected cations uptake compared to chemical fertilizer. It also reduced N, P, and K based chemical fertilizer up to 90%. Crude humins (T2, T3, T4, and T5) and other composts (T6, T7, and T9) can be used as alternative for chemical fertilizers because of their similar effects on maize plants' growth and nutrient uptake. These findings could be validated in future field trials.

## Figures and Tables

**Table 1:** Selected chemical and physical properties of Bekenu series.

Property	Data obtained (0–25 cm)	Standard data range [1] (0–36 cm)
pH_water_	4.41	4.6–4.9
pH_KCl_	3.25	3.8–4.0
CEC (cmol kg^−1^)	11.97	3.86–8.46
Total N (%)	0.08	0.04–0.17
Exchangeable NH_4_^+^ (mg kg^−1^)	21.02	Nd
Available NO_3_^−^ (mg kg^−1^)	7.01	Nd
Available P (mg kg^−1^)	4.85	Nd
Exchangeable K (cmol kg^−1^)	0.10	0.05–0.19
Exchangeable Ca (cmol kg^−1^)	0.25	0.01
Exchangeable Mg (cmol kg^−1^)	0.34	0.07–0.21
Exchangeable Na (cmol kg^−1^)	0.22	0.01
C/N ratio	28.93	14-15
Organic matter (%)	4.19	Nd
Total carbon (%)	2.43	0.57–2.51
Bulk density (g cm^−3^)	1.16	Nd
Sand %	71.04	72–76
Silt %	14.58	8-9
Clay %	14.38	16–19
Texture	SCL	SCL

CEC: cation exchange capacity; Nd: not determined; SCL: sandy clay loam.

**Table 2:** Treatments evaluated in a pot experiment using *Zea mays* L. as test crop.

Treatment	Urea (g)	ERP (g)	K source (g)
T0	0.00	0.00	0.00
T1	4.84	7.45	2.48 MOP
T2	3.24	4.95	53.02 RS humin
T3	3.58	5.67	68.90 RH humin
T4	3.86	6.36	63.22 SD humin
T5	3.94	6.48	43.39 EFB humin
T6	2.92	4.51	50.95 RS compost
T7	0.83	0.00	164.46 RH compost
T8	0.26	1.07	227.48 SD compost
T9	3.71	5.31	53.05 EFB compost

Each treatment from T1 to T9 supplies equivalent nutrients at rate of 2.23 g N, 2.23 g P_2_O_5_, and 1.49 g K_2_O [2].

**Table 3:** Chemical properties of different composts at initial and final stages of composting.

Property	Rice straw		Percentage difference	Rice husk		Percentage difference	Sawdust		Percentage difference	EFB		Percentage difference
	Initial	End		Initial	End		Initial	End		Initial	End	
pH_water_	7.07	7.58^c^ (±0.10)	+7.21	7.33	7.98^b^ (±0.05)	+8.87	7.69	7.64^c^ (±0.04)	−0.65	8.07	9.20^a^ (±0.07)	+14.00
pH_KCl_	7.08	7.14^bc^ (±0.09)	+0.85	6.83	7.35^b^ (±0.07)	+7.61	6.80	6.96^c^ (±0.02)	+2.35	8.33	8.75^a^ (±0.06)	+5.04
Ash (%)	16.67	26.00^a^ (±0.58)	+55.97	20.33	26.33^a^ (±0.33)	+29.51	5.67	9.33^c^ (±0.67)	+64.55	11.00	15.33^b^ (±1.45)	+39.36
Organic matter (%)	83.33	74.00^c^ (±0.58)	−11.20	79.67	73.67^c^ (±0.33)	−7.53	94.33	90.67^a^ (±0.67)	−3.88	89.00	84.67^b^ (±1.45)	−4.87
Total organic carbon (%)	48.33	42.92^c^ (±0.33)	−53.11	46.21	42.73^c^ (±0.19)	−7.53	54.71	52.59^a^ (±0.39)	−3.87	51.62	49.11^b^ (±0.84)	−4.86
Nitrogen (%)	1.06	1.85^a^ (±0.07)	+74.53	1.14	1.21^b^ (±0.02)	+6.14	0.72	0.98^c^ (±0.02)	+36.11	0.66	1.01^c^ (±0.04)	+53.03
Phosphorus (%)	0.47	0.75^a^ (±0.01)	+59.57	0.40	0.59^b^ (±0.00)	+47.5	0.19	0.37^c^ (±0.02)	+94.74	0.22	0.53^b^ (±0.02)	+140.91
C/N ratio	46.19	23.27^c^ (±0.86)	−49.62	40.98	35.22^b^ (±0.71)	−14.06	77.55	53.65^a^ (±0.89)	−30.82	79.79	48.85^a^ (±2.00)	−38.78
C/P ratio	104.87	57.02^c^ (±0.96)	−47.63	115.83	72.27^bc^ (±0.32)	−37.61	287.04	144.80^a^ (±9.75)	−49.55	234.44	93.41^b^ (±3.54)	−60.17
CEC (cmol kg^−1^)	48.87	93.60^a^ (±3.10)	+91.53	45.80	54.07^c^ (±2.07)	+18.06	39.33	76.20^b^ (±0.40)	+93.75	42.13	54.47^c^ (±1.01)	+29.29
Humic acid (%)	4.17	10.97^a^ (±1.73)	+163.07	2.60	3.13^b^ (±0.09)	+20.38	4.20	4.23^b^ (±0.03)	+0.71	3.17	3.27^b^ (±0.03)	+3.15

Different alphabets within a row indicate significant difference between means of compost at the end of composting using Tukey's test at *P* ≤ 0.05.

Positive and negative symbols at the beginning of the number represent the decrease and increase in each items.

( ) values in parenthesis represent standard error of the mean.

**Table 4:** Cation content (mg kg^−1^) in different composts at initial and final stages of composting.

Cation content (mg kg^−1^)	Rice straw		Percentage difference	Rice husk		Percentage difference	Sawdust		Percentage difference	EFB		Percentage difference
	Initial	End		Initial	End		Initial	End		Initial	End	
K	15121	24200^a^ (±201.33)	+60.04	5976	7497^c^ (±20.1)	+25.45	4904	5420^d^ (±77.27)	+10.52	16797	23242^b^ (±191.51)	+38.37
Ca	11182	12013^a^ (±409.69)	+7.43	8181	9996^a^ (±520.84)	+22.19	8536	11236^a^ (±1307.32)	+31.63	7866	12315^a^ (±134.00)	+56.56
Mg	2566	7511^a^ (±199.77)	+192.71	2881	6713^a^ (±80.40)	+133.01	1755	5554^a^ (±941.51)	+216.47	3049	7343^a^ (±409.36)	+140.83
Na	1641	2439^ab^ (±69.95)	+48.63	1521	1950^ab^ (±221.40)	+28.21	1742	1896^b^ (±100.05)	+8.84	1956	2533^a^ (±46.42)	+29.50
Cu	63	71^c^ (±8.61)	+12.70	72	105^b^ (±3.23)	+45.83	118	120^b^ (±1.29)	+1.67	134	159^a^ (±1.30)	+18.66
Zn	140	164^b^ (±8.16)	+17.14	108	147^b^ (±7.85)	+36.11	126	136^b^ (±5.60)	+7.94	161	198^a^ (±2.89)	+22.98
Mn	137	223^b^ (±2.57)	+62.77	187	295^a^ (±14.63)	+57.75	20	90^d^ (±18.52)	+350	46	151^c^ (±4.32)	+228.26
Fe	1833	2541^a^ (±445.20)	+38.63	646	815^b^ (±98.90)	+26.16	509	593^b^ (±87.01)	+16.50	927	1418^b^ (±133.62)	+52.97

Different alphabets within a row indicate significant difference between means of compost at the end of composting using Tukey's test at *P* ≤ 0.05.

Positive and negative symbol at the beginning of the number is represents the decrease and increase in each items.

( ) values in parenthesis represent standard error of the mean.

**Table 5:** Chemical properties of humic acids from different composts.

Property	Rice straw	Rice husk	Sawdust	EFB	Tan (2003) [3]
*E*_4_/*E*_6_	7.18^a^ (±0.07)	6.91^ab^ (±0.08)	6.15^d^ (±0.07)	6.62^c^ (±0.05)	7-8
Carbon (%)	55.29^b^ (±0.39)	55.29^b^ (±0.39)	56.84^a^ (±0.00)	56.07^ab^ (±0.39)	56–62
Phenolic (cmol kg^−1^)	250.00^a^ (±0.00)	233.33^a^ (±33.33)	233.33^a^ (±33.33)	200.00^a^ (±0.00)	240–540
Carboxylic (cmol kg^−1^)	366.67^ab^ (±8.33)	358.33^ab^ (±8.33)	383.33^a^ (±8.33)	341.67^b^ (±8.33)	150–440
Total acidity (cmol kg^−1^)	616.67^a^ (±8.33)	591.67^a^ (±30.05)	616.67^a^ (±30.05)	541.67^a^ (±8.33)	500–700
Total K (%)	0.250^a^ (±0.03)	0.131^b^ (±0.02)	0.267^a^ (±0.03)	0.209^ab^ (±0.02)	nd
Total Ca (%)	0.054^b^ (±0.00)	0.050^b^ (±0.00)	0.081^a^ (±0.00)	0.056^b^ (±0.00)	nd
Total Mg (%)	0.020^b^ (±0.00)	0.065^a^ (±0.00)	0.019^b^ (±0.00)	0.012^b^ (±0.00)	nd
Total Na (%)	0.223^a^ (±0.03)	0.201^a^ (±0.01)	0.248^a^ (±0.02)	0.220^a^ (±0.01)	nd

Different alphabets within a row indicate significant difference between means using Tukey's test at *P* ≤ 0.05.

( ) values in parenthesis represent standard error of the mean.

**Table 6:** pH and major cation contents (mg kg^−1^) of crude fulvic acids from different composts.

Property	Rice straw	Rice husk	Sawdust	EFB
pH	1.59^a^ (±0.01)	1.53^b^ (±0.01)	1.54^b^ (±0.00)	1.55^b^ (±0.01)
Total K (%)	7.45^a^ (±0.08)	4.58^c^ (±0.22)	3.82^d^ (±0.13)	5.56^b^ (±0.18)
Total Ca (%)	3.63 × 10^−3^ ^a^ (±1.1 × 10^−5^)	2.71 × 10^−4^ ^d^ (±4.0 × 10^−6^)	1.88 × 10^−3^ ^b^ (±7.0 × 10^−6^)	1.38 × 10^−3^ ^c^ (±6.0 × 10^−6^)
Total Mg (%)	2.07 × 10^−2^ ^a^ (±5.9 × 10^−5^)	8.29 × 10^−3^ ^c^ (±3.2 × 10^−5^)	1.93 × 10^−2^ ^b^ (±5.9 × 10^−5^)	6.12 × 10^−3^ ^d^ (±7.4 × 10^−5^)
Total Na (%)	0.146^a^ (±0.0006)	0.091^c^ (±0.0003)	0.074^d^ (±0.0006)	0.095^b^ (±0.0006)

Different alphabets within a row indicate significant difference between means using Tukey's test at *P* ≤ 0.05.

( ) values in parenthesis represent standard error of the mean.

**Table 7:** Chemical properties of crude humins from different composts.

Property	Rice straw	Rice husk	Sawdust	EFB
pH_water_	9.42^d^ (±0.01)	9.63^c^ (±0.02)	9.80^b^ (±0.01)	9.86^a^(±0.01)
pH_KCl_	9.22^d^ (±0.00)	9.40^c^ (±0.01)	9.45^b^ (±0.01)	9.81^a^ (±0.01)
OM (%)	73.33^c^ (±1.20)	75.00^c^ (±0.58)	90.33^a^ (±0.88)	84.67^b^ (±1.33)
TOC (%)	42.53^c^ (±0.70)	43.50^c^ (±0.33)	52.39^a^ (±0.51)	49.11^b^ (±0.77)
CEC (cmol kg^−1^)	62.00^a^ (±3.86)	46.07^b^ (±1.10)	52.60^ab^ (±1.90)	46.07^b^ (±1.27)
Total N (%)	1.39^a^ (±0.05)	0.84^bc^ (±0.03)	0.72^c^ (±0.04)	0.95^b^ (±0.02)
Total P (%)	0.62^a^ (±0.02)	0.34^b^ (±0.00)	0.23^c^ (±0.01)	0.30^b^ (±0.02)

Different alphabets within a row indicate significant difference between means using Tukey's test at *P* ≤ 0.05.

( ) values in parenthesis represent standard error of the mean.

**Table 8:** Total and exchangeable cations (mg kg^−1^) in crude humins from different composts.

Cations	Rice straw	Rice husk	Sawdust	EFB
K (%)	2.33^b^ (±0.06)	1.79^c^ (±0.06)	1.95^c^ (±0.02)	2.84^a^ (±0.08)
Ca (%)	1.46^a^ (±0.16)	1.03^ab^ (±0.11)	0.76^b^ (±0.06)	1.44^a^ (±0.09)
Mg (%)	0.79^a^ (±0.08)	0.53^bc^ (±0.05)	0.33^c^ (±0.03)	0.73^ab^ (±0.06)
Na (%)	0.18^a^ (±0.00)	0.14^b^ (±0.01)	0.17^a^ (±0.01)	0.18^a^ (±0.01)
Fe (%)	0.21^a^ (±0.01)	0.07^c^ (±0.00)	0.05^c^ (±0.00)	0.13^b^ (±0.01)
Cu (mg kg^−1^)	39.70^c^ (±2.23)	29.43^d^ (±2.23)	86.53^b^ (±1.21)	97.50^a^ (±2.82)
Mn (mg kg^−1^)	226.60^a^ (±16.93)	232.83^a^ (±15.87)	46.57^c^ (±6.42)	148.50^b^ (±6.63)
Zn (mg kg^−1^)	164.43^b^ (±5.27)	81.33^d^ (±2.65)	117.60^c^ (±1.21)	184.93^a^ (±3.63)
Exchangeable K (cmol kg^−1^)	38.25^a^ (±1.16)	28.95^b^ (±0.44)	25.79^b^ (±1.83)	37.57^a^ (±1.01)
Exchangeable Ca (cmol kg^−1^)	12.58^b^ (±0.46)	12.60^b^ (±0.05)	12.45^b^ (±1.13)	21.50^a^ (±0.53)
Exchangeable Mg (cmol kg^−1^)	6.96^b^ (±0.16)	6.81^b^ (±0.09)	9.50^a^ (±0.58)	10.04^a^ (±0.20)
Exchangeable Na (cmol kg^−1^)	4.11^a^ (±0.09)	3.10^b^ (±0.04)	3.14^b^ (±0.21)	3.55^b^ (±0.08)

Different alphabets within a row indicate significant difference between means using Tukey's test at *P* ≤ 0.05.

( ) values in parenthesis represent standard error of the mean.

**Table 9:** Selected soil chemical properties at 48 DAS.

Property	T0	T1	T2	T3	T4	T5	T6	T7	T8	T9
pH_water_	4.81^c^ (±0.06)	4.51^d^ (±0.03)	5.01^b^ (±0.03)	5.20^a^ (±0.04)	5.05^ab^ (±0.02)	4.80^c^ (±0.02)	4.69^c^ (±0.03)	5.01^b^ (±0.02)	5.06^ab^ (±0.02)	5.13^ab^ (±0.03)
OM (%)	4.00^d^ (±0.14)	4.25^cd^ (±0.17)	4.35^cd^ (±0.17)	4.75^bcd^ (±0.15)	5.00^bc^ (±0.22)	4.55^bcd^ (±0.10)	4.55^bcd^ (±0.25)	5.40^b^ (±0.23)	7.95^a^ (±0.32)	4.65^bcd^ (±0.15)
Total carbon (%)	2.32^d^ (±0.08)	2.47^cd^ (±0.10)	2.52^cd^ (±0.10)	2.76^bcd^ (±0.09)	2.90^bc^ (±0.13)	2.64^bcd^ (±0.06)	2.64^bcd^ (±0.15)	3.13^b^ (±0.13)	4.61^a^ (±0.19)	2.70^bcd^ (±0.09)
Total N (%)	0.11^c^ (±0.01)	0.12^bc^ (±0.01)	0.13^abc^ (±0.01)	0.12^bc^ (±0.01)	0.13^abc^ (±0.01)	0.11^c^ (±0.00)	0.13^abc^ (±0.01)	0.15^ab^ (±0.01)	0.16^a^ (±0.01)	0.13^abc^ (±0.01)
Exchangeable NH_4_^+^ (mg kg^−1^)	40.28^b^ (±5.98)	99.83^a^ (±12.59)	22.77^b^ (±5.26)	22.77^b^ (±6.63)	35.03^b^ (±2.86)	21.02^b^ (±2.86)	19.27^b^ (±3.35)	17.52^b^ (±2.02)	33.28^b^ (±3.35)	21.02^b^ (±2.86)
Available NO_3_^−^ (mg kg^−1^)	22.77^bc^ (±1.75)	21.02^bc^ (±2.86)	26.27^bc^ (±5.98)	19.27^bc^ (±1.75)	24.52^bc^ (±2.02)	29.77^bc^ (±5.98)	17.52^c^ (±2.02)	15.76^c^ (±1.75)	47.29^a^ (±3.35)	35.03^ab^ (±2.86)
Available P (mg kg^−1^)	1.68^d^ (±0.23)	49.74^a^ (±3.83)	30.36^cb^ (±2.92)	48.58^a^ (±4.43)	47.84^ab^ (±3.77)	50.96^a^ (±5.59)	28.91^c^ (±3.68)	30.74^bc^ (±1.89)	37.82^abc^ (±2.30)	39.26^abc^ (±4.29)
Exchangeable K (cmol kg^−1^)	0.09^d^ (±0.00)	0.29^bc^ (±0.03)	0.28^bc^ (±0.05)	0.63^a^ (±0.03)	0.27^bc^ (±0.01)	0.30^bc^ (±0.03)	0.29^bc^ (±0.01)	0.38^b^ (±0.00)	0.25^c^ (±0.02)	0.34^bc^ (±0.01)
CEC (cmol kg^−1^)	10.93^bc^ (±0.43)	9.80^c^ (±0.28)	12.05^ab^ (±0.31)	11.50^abc^ (±0.34)	11.58^abc^ (±0.46)	11.83^abc^ (±0.38)	12.53^ab^ (±1.07)	13.30^a^ (±0.26)	13.73^a^ (±0.19)	11.70^abc^ (±0.32)

Different letters within a row indicate significant difference between means using Tukey's test at *P* ≤ 0.05.

( ) values in parenthesis represent standard error of the mean.
